# Contemporary Antiretroviral Therapy Regimens in People With HIV Who Initiated Treatment in the Pre‐Antiretroviral Therapy Era

**DOI:** 10.1002/jmv.70708

**Published:** 2025-11-14

**Authors:** Eisuke Adachi, Shuhei Okuno, Yoshiaki Kanno, Michiko Koga, Hiroshi Yotsuyanagi

**Affiliations:** ^1^ Department of Infectious Diseases and Applied Immunology IMSUT Hospital of The Institute of Medical Science, The University of Tokyo Tokyo Japan

## Author Contributions

Eisuke Adachi designed and conducted the study and was responsible for writing the manuscript. Eisuke Adachi, Shuhei Okuno, Yoshiaki Kanno, Michiko Koga, and Hiroshi Yotsuyanagi extracted clinical data, including characteristics of PWH and ART regimens, from the in‐house database, which were subsequently curated by Eisuke Adachi.

## Ethics Statement

Ethics approval was granted by the ethics board of the Institute of Medical Science, University of Tokyo (2022‐48‐1128).

## Conflicts of Interest

Eisuke Adachi has received honoraria for lectures and advisory board participation from ViiV Healthcare K.K., Tokyo, Japan, ViiV Healthcare, London, UK, GlaxoSmithKline, Brentford, UK, and Gilead Sciences K.K., Tokyo, Japan, and Gilead Sciences Inc., Foster City, CA, USA.

To the Editor,

The treatment of HIV has evolved dramatically since the pre‐antiretroviral therapy (ART) era, when monotherapy with zidovudine (AZT) or dual nucleoside reverse transcriptase inhibitors (NRTIs) therapy was commonly used [1]. A major milestone was the introduction of combination ART in 1996, consisting of two NRTIs plus a key drug, typically a protease inhibitor (PI). Currently, integrase strand transfer inhibitors‐based regimen, such as dolutegravir/lamivudine (DTG/3TC) or bictegravir/emtricitabine/tenofovir alafenamide (B/F/TAF), as well as long‐acting antiretroviral formulations like cabotegravir plus rilpivirine (CAB + RPV), can suppress HIV even in the presence of historical resistance mutations. Nevertheless, lamivudine (3TC) and emtricitabine (FTC) remain widely used, and resistance to these agents is still common [2]. Against this backdrop, we aimed to characterize current ART regimens among heavily treatment‐experienced (HTE) people with HIV (PWH), focusing on individuals with over 30 years of continuous ART history.

We retrospectively analyzed PWH who initiated antiretroviral drugs (ARVs) at IMSUT Hospital, Institute of Medical Science, The University of Tokyo, before 1996, and continued from April 2019 onwards. Eligible individuals had available HIV RNA data from both early ARVs, and the initiation of an effective three‐drug regimen (3DR), and with confirmed virologic outcomes and the most recent ART regimens since April 2019, when B/F/TAF became available in Japan. Clinical data collected included age, the year of initial ARV initiation, HIV RNA levels at the start of effective 3DR, the presence of M184V/I mutations, history of ARV use, and the longitudinal history of ART regimens up to April 2025.

Among 202 individuals who initiated ARVs before 1996, 23 remained in follow‐up after April 2019. The median current age in 2025 was 64 years (range, 50–82). Initial regimens consisted of NRTI monotherapy in 8 individuals (6 with AZT) and dual NRTI therapy in 14 individuals (Table 1). Seventeen individuals (74%) had detectable viremia at the time of switching to two NRTIs plus a key drug after 1996, with a median HIV RNA of 19,500 copies/mL (range, 740–170,000), while six achieved virologic suppression even before the ART era (Table 2). The M184V mutation had been detected in 7 individuals (30%), and nearly half had prior exposure to 3TC with subsequent virological failure.

**Table 1:** Distribution of Initial Antiretroviral Agents and Current ART Regimens.

Total, N				23
ARVs at Initiation in the Pre‐ART Era	NRTI monotherapy		AZT	6
			ddi	2
	2NRTIs		AZT+ddi	7
			AZT + 3TC	6
			AZT+ddC	1
	NRTI + PI		AZT + IDV	1
ART Regimen at Most Recent Visit in the ART Era*	TAF‐based 3DR	STR	TAF/FTC/BIC	5
			TAF/FTC/DRV/cobi	1
			TAF/FTC/RPV	1
		MTR	TAF/FTC + DTG	1
			TAF/FTC/DRV/cobi+DOR	1
	2DR	STR	DTG/3TC	7
			RPV/DTG	2
		MTR	DTG + DRV/cobi	1
			DRV/cobi+RAL	1
		Injectable	CAB + RPV	1
	others		DRV/cobi+DOR + DTG/3TC	1
			TDF + DTG/RPV	1

Abbreviations: 2DR, two‐drug regimen; 3DR, three‐drug regimen; 3TC, lamivudine; AZT, zidovudine; BIC, bictegravir; cobi, cobicistat; DOR, doravirine; ddC, zalcitabine; ddI, didanosine; DRV, darunavir; DTG, dolutegravir; FTC, emtricitabine; IDV, indinavir; MTR, multiple‐tablet regimen; RAL, raltegravir; RPV, rilpivirine; STR, single‐tablet regimen; TAF, tenofovir alafenamide; TDF, tenofovir disoproxil fumarate.

*Most recent visit indicates the most recent regimen prescribed after April 2019.

**Table 2:** Clinical and antiretroviral treatment characteristics of 23 heavily treatment‐experienced individuals.

Case No.	Current age^a^	Year of first ARV administration	ARVs in the pre‐ART era	ART history in the ART era^b^	VF on 3TC in the pre‐ART era	HIV‐RNA at start of effective ART, copies/mL
1	mid‐60s	1988	AZT; ddI; AZT + ddI;	AZT + ddC + IDV; AZT + 3TC + IDV; d4T + 3TC + NFV; RTV + SQV; d4T + 3TC + RTV + SQV; AZT+ddI+RTV + SQV; AZT + ddI + RTV + SQV; AZT + ddI + RTV + SQV + EFV; ddI + 3TC + ABC + APV + RTV + EFV; ddI+3TC + ABC + APV + RTV + EFV; ddI + ABC/3TC + fAPV + RTV + EFV; TAF/FTC + DRV/cobi + EFV; TAF/FTC/DRV/cobi + EFV; TAF/FTC/DRV/cobi + DOR	Yes	50,000
2	mid‐70s	1992	AZT; AZT + ddI; AZT + 3TC;	ddI + LPV/RTV + EFV; ddI + LPV/RTV + NVP; ETR + DRV + RTV + RAL; ETR + DRV/cobi+RAL; DTG + DRV/cobi	Yes	22,000
3	early 60 s	1992	AZT	d4T + 3TC + NFV; AZT/3TC + EFV; TDF/FTC + EFV; TDF/FTC + DRV + RTV; TDF/FTC + DTG; TAF/FTC + DTG; DTG/3TC	No	99,000
4	late 50 s	1994	AZT; AZT + ddI; AZT + ddC	AZT + ddC + IDV; d4T + 3TC + NFV; TDF/FTC + ddI + LPV/RTV; TDF/FTC + LPV/RTV + RAL; RPV + DTG; RPV/DTG	Yes	< 400‡
5	mid‐60s	1994	AZT; ddI; AZT + ddI; AZT + 3TC; SQV + ddC	AZT + ddC + SQV; AZT + ddI + IDV; ABC + 3TC + EFV + RTV + SQV; 3TC + ddI + RTV + SQV; 3TC + ddI + RTV + SQV + NVP; 3TC + ddI + fAPV + RTV; ddI + 3TC + SQV + RTV; 3TC + NVP + RAL; TDF/FTC + NVP + RAL; TAF/FTC + NVP + RAL; TDF/FTC + DTG; DTG + DRVcobi; TAF/FTC/BIC	Yes	20,000
6	early 70 s	1995	AZT + ddI; AZT + 3TC	AZT + ddC + IDV; d4T + 3TC + NVP; TDF + 3TC + EFV; TDF + 3TC + ATV + RTV; TDF/FTC + ATV + RTV; TDF/FTC + RAL; ABC/3TC + RAL; ABC/3TC + DTG; ABC/3TC/DTG; DTG/3TC	No	79,000
7	mid‐60s	1995	AZT + ddI	d4T + 3TC + RTV + SQV; d4T + 3TC + EFV; AZT + 3TC + CBV + EFV; TDF/FTC + ATV + RTV; ABC/3TC + ATV + RTV; ABC/3TC + DRV + RTV; ABC/3TC + DTG; ABC/3TC + DTG; CAB + RPV	No	58,000
8	early 60 s	1995	AZT; AZT + ddI; AZT + ddC	AZT + ddC + IDV; ABC + 3TC + EFV + LPV/RTV; ABC + 3TC + LPV/RTV + RAL; LPV/RTV + ABC/3TC/DTG; DRV + RTV + ABC/3TC/DTG; TAF/FTC/BIC	No	3700
9	early 70 s	1996	AZT + ddI; AZT + ddC	d4T + 3TC + NFV; d4T + 3TC + NFV(BID); TDF/FTC + ATV + RTV; TDF/FTC + DRV + RTV; TDF/FTC + RPV; TAF/FTC/RPV	No	3000
10	late 60 s	1995	AZT + ddI; AZT + ddC	d4T + 3TC + NFV; d4T + 3TC + NFV(BID); TDF + 3TC + ATV + RTV; TDF/FTC + ATV + RTV; TDF/FTC + DTG; ABC/3TC + DTG; ABC/3TC/DTG; ABC/3TC/DTG; ABC/3TC/DTG; DTG/3TC	No	170,000
11	early 60 s	1996	AZT + 3TC; AZ + ddC	AZT + ddC + NFV; ABC/3TC + EFV; ABC/3TC + DTG; ABC/3TC/DTG; DTG/3TC	Yes	< 400^c^
12	early 70 s	1996	AZT + ddI	AZT + ddC + SQV; d4T + 3TC + NFV; ABC/3TC + ATV + RTV; TDF/FTC + ATV + RTV; TDF/FTC + EVG/COBI; TAF/FTC + EVG/COBI; TAF/FTC/BIC	No	740
13	mid‐60s	1995	AZT + ddI; d4T + 3TC	ATV + RTV + TDF + 3TC + EFV; TDF/FTC + ATV + RTV; DRV + RTV; TAF/FTC + DTG; TAF/FTC/BIC	Yes	8300
14	early 80 s	1996	AZT + ddI	d4T + 3TC + LPV/rtv; AZT/3TC + LPV/rtv; ABC/3TC + LPV/rtv; ABC/3TC + DRV+rtv; ABC/3TC + DTG; DTG/3TC	No	2900
15	mid‐50s	1996	ddI AZT + ddC AZT + 3TC	d4T+RTV + SQV; d4T+ddI+NFV + HU d4T+NFV; ABC + EFV + LPV/rtv; RAL + LPV/rtv; RPV + RAL; RPV + DTG; RPV/DTG	Yes	27,000
16	early 70 s	1996	AZT + 3TC	DTG + AZT/3TC; RPV + DTG; DRV/rtv+DTG; DRV/rtv+RAL; DRV/cobi+RAL	0 No	< 400^c^
17	late 50 s	1996	AZT + 3TC; ddC+IDV	d4T + 3TC + NFV; TDF + 3TC+fAPV+rtv; TDF + 3TC + DTG; TAF/FTC + DTG; TAF/FTC/BIC	Yes	6700
18	early 70 s	1996	AZT	ABC/3TC + RAL; ABC/3TC + DTG; ABC/3TC/DTG; TAF/FTC + BIC; DTG/3TC	No	< 400^a^
19	late 50 s	1996	AZT + 3TC	ABC/3TC + RAL; AZT + 3TC + RAL; TAF/FTC + DTG	No	< 400^c^
20	early 60 s	1996	AZT + 3TC; AZT + 3TC	d4T + NFV + EFV; d4T + ddI + EFV; AZT + 3TC + TDF + ATV+rtv; AZT + 3TC + TDF + fAPV + rtv; TDF/FTC + AZT+fAPV + rtv; TDF/FTC + AZT + RAL; ABC/3TC + AZT + RAL; TDF + RPV + DTG; TDF + RPV/DTG	Yes	19,000
21	late 50 s	1996	AZT + IDV	d4T + 3TC + NFV; ddI+HU + EFV + APV; ddI+EFV + APV; AZT/3TC + LPV/rtv + SQV; AZT/3TC + LPV/rtv; TDF + 3TC + EFV + ATV+rtv; AZT/3TC + TDF + LPV/rtv; T20 + TPV + ABC + rtv; T20 + AZT + TDF/FTC + LPV/rtv; T20 + AZT + TDF/FTC; AZT + TDF/FTC + LPV/rtv; AZT + TDF/FTC+fAPV+rtv+EFV; DRV + TMC125 + MK‐0518 + 3TC + rtv; DRV + TMC125 + RAL + 3TC + rtv; DRV + ETR + RAL + 3TC + rtv; DRV + ETR + DTG + 3TC + rtv; DRV + ETR + 3TC/DTG + rtv; DRV/cobi + ETR + DTG/3TC; DRV/cobi+DOR + DTG/3TC	Yes	16,000
22	early 50 s	1996	AZT + ddC; AZT + 3TC	ABC/3TC + RAL; ABC/3TC/DTG; DTG/3TC	No	< 400
23	mid‐70s	1996	AZT + 3TC	d4T + SQV + NFV; d4T + NFV + SQV; TDF + EFV + ATV + rtv; TDF + 3TC + EFV + ATV + rtv; TDF + AZT/3TC + ATV + rtv; TDF + AZT/3TC + DRV + rtv; TDF + RPV + DRV + rtv; RPV + DRV + rtv + DTG; DRV + rtv + DTG; DRV/cobi + DTG; TAF/FTC/DRV/cobi	Yes	940

Abbreviations: 3TC, lamivudine; ABC, abacavir; APV, amprenavir; ARV, antiretroviral; ART, antiretroviral therapy; ATV, atazanavir; AZT, zidovudine; BIC, bictegravir; cobi, cobicistat; d4T, stavudine; ddC, zalcitabine; ddI, didanosine; DOR, doravirine; DRV, darunavir; DTG, dolutegravir; EFV, efavirenz; ETR, etravirine; fAPV, fosamprenavir; FTC, emtricitabine; HU, hydroxyurea; LPV, lopinavir; MK‐0518, raltegravir (development code); NFV, nelfinavir; RAL, raltegravir; RPV, rilpivirine; rtv, ritonavir; SQV, saquinavir; T20, enfuvirtide; TAF, tenofovir alafenamide; TDF, tenofovir disoproxil fumarate; TMC125, etravirine (development code); TPV, tipranavir; VL, viral load.

^a^ Age as of April 2025.

^b^ Treatment regimen history after initiation of two NRTIs plus a key drug (most commonly a protease inhibitor).

^c^ Below the detection limit of the assay at that time.

Since 2019, the most recent regimens included TAF‐based regimens in nine individuals and two‐drug regimens (2DR) in 12 individuals. B/F/TAF was the most common TAF‐based regimen, while DTG/3TC was the predominant 2DR, with one individual who received CAB + RPV (Table 1). Despite diverse treatment histories and prior resistance, all individuals maintained virologic suppression from April 2019 onward.

In our cohort, the M184V mutation was detected in 30% of individuals. This relatively low detection rate may reflect the limited availability of resistance testing at that time, as commercial assays were not routinely performed. In addition, most pre‐ART era regimens involved AZT, which is not associated with M184V, and none received 3TC monotherapy. These factors likely contributed to the lower observed prevalence [3]. Nevertheless, some individuals experienced viremia on 3TC without any documented resistance mutations, suggesting that 3TC alone was insufficient to fully suppress viral replication.

Regarding current regimens, approximately 70% of individuals were on STR, about 40% received TAF‐based regimens, and nearly 50% were on 2DR. Overall, most individuals used contemporary standard therapies such as B/F/TAF or DTG/3TC, with a subset on CAB + RPV. These findings suggest that even HTE PWH continue to access ART options with current standard‐of‐care practices [4, 5].

3TC and FTC remain widely used, and M184V/I is still the most frequent NRTI‐ associated mutation. Evidence from Study 4030 shows that B/F/TAF maintains viral suppression even in the presence of M184I [2]. In our cohort, B/F/TAF remained active, indicating that M184I alone may not confer clinically significant resistance. Previous pooled analyses of six clinical trials with over 2000 virologically suppressed participants found pre‐existing M184V/I in approximately 10% of cases [6]. Importantly, even when M184V/I coexisted with other resistance‐associated mutations, switching to B/F/TAF achieved durable viral suppression in more than 98% of participants, without emergent resistance [6]. Importantly, these data were limited to virologically suppressed individuals already; the impact in treatment‐naïve individuals remains less clear.

This study has limitations. Of 202 individuals who initiated ARVs during the pre‐ART era, only 23 individuals remained in follow‐up, raising the possibility of survivor bias. HIV RNA quantification was not available until 1996, and the records of ARV use before that may be incomplete. Nevertheless, this cohort provides rare longitudinal data spanning three decades of continuous therapy in HTE individuals. Regarding treatment options for HTE, as of August 2025, lenacapavir is available in Japan, whereas ibalizumab and fostemsavir remained unapproved [7, 8, 9].

In summary, despite drug resistance and extensive treatment histories, these individuals are generally able to maintain virologic suppression with modern ART, underscoring the durability and effectiveness of contemporary regimens in HTE populations.

## Data Availability

The data that support the findings of this study are available on request from the corresponding author. The data are not publicly available due to privacy or ethical restrictions.
